# Effect of Extraction Methods and Preheat Treatments on the Functional Properties of Pumpkin Seed Protein Concentrate

**DOI:** 10.1021/acsfoodscitech.4c00601

**Published:** 2024-12-25

**Authors:** Ozan Tas, S. Gulum Sumnu, Mecit Halil Oztop

**Affiliations:** Department of Food Engineering, Middle East Technical University, Ankara 06800, Turkey

**Keywords:** extraction, microwave, pumpkin seed protein concentrate (PSPC), FTIR spectroscopy, water solubility index (WSI), TD-NMR relaxometry, functional properties

## Abstract

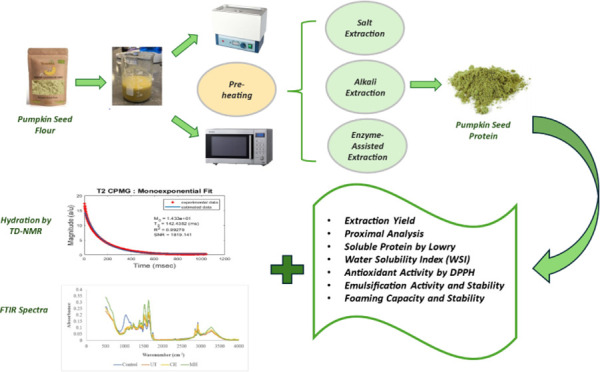

This study explores the effect of different extraction methods and preheat treatments in obtaining protein concentrate from pumpkin seed flour. The effects on the yield and functional properties of pumpkin seed protein concentrate (PSPC) were compared alongside microwave and conventional preheating methods using alkali, salt, and enzyme-assisted alkali extraction techniques. Analytical assessments included proximate analysis, soluble protein content, water solubility index (WSI), emulsification activity (EA) and stability (ES), foaming capacity (FC) and stability (FS), and antioxidant activity (AA). Hydration and structural analyses were performed via time-domain nuclear magnetic resonance (TD-NMR) Relaxometry and Fourier-Transform Infrared (FTIR) Spectroscopy. In addition, color measurements were performed to evaluate the visual quality of the samples. The alkali extraction method paired with microwave heating (MH-AE) significantly outperformed other techniques, with an extraction yield and protein content of approximately 55% and 77%, respectively. This study demonstrated the superior yield and functional properties of PSPC using MH-AE, opening opportunities for future research in optimizing plant-based protein extraction techniques.

## 1 Introduction

People are becoming more attracted to plant proteins as a result of the negative environmental implications of animal protein production as well as expanding veganism and vegetarianism trends.^1^ Furthermore, the food industry is particularly interested in the production of plant protein concentrates or isolates due to their capacity to enhance both the nutritional value and functional properties such as emulsification, foaming, and antioxidant activity.^2^ Protein sources, including peanuts, peas, pumpkin seeds, sesame, lentils, beans, and chickpeas are being widely studied for their nutritional and functional properties.^3-5^ However, despite the growing popularity of these proteins, some drawbacks still remain. Peanuts are a major allergen and may trigger severe allergic responses, which makes them unsuitable for use in several food applications.^6^ Despite being abundant in protein, peas and lentils frequently have strong off tastes and include antinutritional ingredients like tannins and phytic acid, which can make them less palatable and accepted in food products.^7^ Similarly, compounds found in beans and chickpeas, such as oligosaccharides and protease inhibitors, may affect digestion and contribute to gastrointestinal discomfort.^8,9^ Sesame seeds have high oil content,^10^ which can make the extraction of their protein more difficult.

Pumpkin seeds, on the other hand, offer several advantages over other sources. Pumpkin seed (*Cucurbita pepo*) is an edible part of the pumpkins that is produced as a byproduct of pumpkin processing.^11^ It is high in calories and nutrition, with an especially high quantity of fat (mainly linoleic acid and oleic acid), protein (∼35%), dietary fiber, and other numerous micronutrients.^12^ Defatted pumpkin seed flour is rich in protein and contains various protein fractions, including alkali-soluble glutenin, salt-soluble globulin, alcohol-soluble prolamin, and water-soluble albumin.^13,14^ They are not only a rich source of protein and essential fatty acids but are also widely regarded as hypoallergenic, making them suitable for a wider range of consumer products, particularly for individuals with severe allergies.^15^ Additionally, compared to legumes, pumpkin seeds contain lower levels of antinutritional factors and higher amounts of antioxidants, making them an excellent option for functional food applications.^16-18^ Despite these promising attributes, further research is needed on pumpkin seeds due to their potential as a desirable and sustainable protein source as well as their availability as a byproduct of pumpkin processing.

The method utilized in protein extraction has a crucial impact on plant proteins’ composition and functional properties such as soluble protein, antioxidant activity, emulsifying, and foaming properties.^19,20^ However, extracting proteins from plant sources presents certain obstacles, such as the presence of high fiber content, sticky structures, or tough cellular components like cell walls.^21^ To overcome these challenges, many methods are used to extract plant proteins from their flours, including extraction with alkali, salt, or enzymes.^22^

Alkali extraction is a common approach for protein extraction that occurs with protein solubilization at alkaline pH (>7), followed by centrifugation to remove insoluble particles, resulting in the proteins eventually precipitating at their isoelectric points.^23^ In salt extraction, the proteins are dissolved in a salt solution and the globulin proteins dissociate into their subunits.^24^ The salt extraction method utilizes the “salting in” and “salting out” properties of salts to facilitate protein extraction. “Salting in” enhances protein solubility, whereas “salting out” reduces both the solubility of proteins and the overall yield of extracted proteins.^25^ Utilizing enzymes is another technique for protein extraction. Protease pretreatment is a common approach for assisting the enzyme in protein extraction.^26^ Especially, the high activity of ***alcalase*** for protein extraction was shown as an effective protease in previous studies.^27−29^

Studies have explored different extraction methods on plant proteins and their effects on functional properties. Gao et al.^30^ showed that alkali extraction could enhance the solubility and emulsifying properties of proteins obtained from legumes, although they noted a risk of protein denaturation at high pH. Similarly, Miranda et al.^31^ compared enzyme-assisted extraction (EE) and alkali extraction (AE) for lentil proteins and demonstrated that EE resulted in improved protein functionality. The studies have also focused on the combined effects of alkali, salt, and enzyme extraction in conjunction with other applications, such as microwave^32,33^ and ultrasound,^34^ to improve protein extraction yield. Microwave application involves simultaneous heat and mass transfer from the interior part of the solid matrix to the extraction solvent. Furthermore, treatment time is significantly shortened when compared to conventional heated extraction methods.^35,36^ The extraction yields of the analyte with microwave in the methodology are comparable to or even higher than those achieved using traditional methods but with reduced solvent consumption and shorter extraction times.^37^ Investigations into microwave-assisted extraction (MAE) by Amponsah et al.^38^ revealed that MAE enhanced extraction efficiency and improved the properties of soy proteins more than traditional heating, with less energy consumption and shorter processing times.

The power level and duration of the microwave are important factors that affect the quality and efficiency of protein extraction from plant materials.^39,40^ Microwave power influences the solubility and release of proteins and influences the rates at which heat is generated in the sample matrix.^41^ Higher power levels have the potential to accelerate heating and improve protein solubilization by increasing the penetration of microwave energy. However, overpowering can denature the proteins and reduce their functional properties.^42^

Several studies in the literature have reported the extraction of pumpkin seed protein using techniques such as ultrasound-assisted extraction and ultrasound–microwave synergistic extraction (UMSE). Das et al.^43^ performed ultrasonic treatment with alkaline extraction in their study to improve the functional properties of the extracted protein, while Liu et al.^44^ showed an alternative method with a UMSE approach to enhance protein yield. However, these studies did not optimize the pretreatment conditions or explore different extraction methods. In contrast, to enhance the nutritional and functional qualities of pumpkin seed protein concentrate (PSPC) and address sustainability concerns, this study aims to optimize the extraction process by examining the effects of different extraction methods and preheat treatments on the characteristics of PSPC.

The hypothesis driving this study proposed that different extraction techniques would have distinct impacts on PSPC characteristics. Furthermore, it was hypothesized that microwave pretreatment would enhance PSPC yield, protein content, and functional properties such as solubility, antioxidant, emulsifying, and foaming activities more effectively than conventional heating methods. This research aims to make a significant contribution to the field by identifying superior methods for producing high-quality plant-based protein concentrates and addressing the growing demand for sustainable protein sources in the food industry. By introducing a novel approach to PSPC extraction through various methods, this study fills a gap in the current literature and contributes to the optimization of its production.

## 2 Materials and Methods

### 2.1 Materials

Defatted pumpkin seed (*Cucurbita pepo*) flour was sourced from Tazemiz (Mersin, Turkey). The enzyme Alcalase (2.4 L), which has a declared activity of 2.4 AU (Anson units) per gram, was obtained from Novozymes (Bagsvaerd, Denmark). All other chemicals and reagents, including solvents and buffers, were purchased from Sigma-Aldrich Co. (St. Louis, MO, USA).

### 2.2 Extraction of Pumpkin Seed Proteins

#### 2.2.1 Alkali Extraction

First, pumpkin seed flour was mixed with water in a ratio of 1:10 (w/v). The pH of this solution was set to 11 by using 1 M NaOH. For the microwave pre-heating, this solution was put into the microwave oven (Kenwood, New Jersey, USA) with a power of 416 W until the temperature reached 50 °C (∼50 s). In the case of conventional pre-heating, the solution was put into the water bath until the temperature reached 50 °C (∼15 min). The solution was then shaken in an orbital shaker (Daihan Scientific Co., Ltd., Korea) at 100 rpm for 1 h and subsequently centrifuged at 2263*g* for 15 min. The resulting supernatant, containing soluble protein, was adjusted to pH 5 (the isoelectric point of the protein) by using 1 M HCl to precipitate the proteins. These precipitated proteins were recovered by centrifugation at 2263*g* for 15 min. Finally, the proteins were dried by using a lyophilizer (Beijing Songyuan Huaxing Technology Development Co., Ltd., China) for 36 h. The same procedure for extracting untreated samples was followed, excluding the preheating steps.

#### 2.2.2 Salt Extraction

The method of Onsaard^45^ was followed with a slight modification. Pumpkin seed flour was mixed with 1 M NaCl at pH 7 in a ratio of 1:10 (w/v), following the same preheating conditions mentioned earlier. The resulting mixture was stirred for 1 h and then centrifuged at 2263*g* for 15 min. The supernatant was collected, and its pH was adjusted to 5 by using 1 M HCl to precipitate the proteins. The mixture was centrifuged again at 2263*g* for 15 min, after which the supernatant was discarded, and the protein-containing precipitate was collected. The proteins were neutralized, as previously described, and subsequently lyophilized. This procedure was repeated for the untreated samples, with the only modification being the omission of the preheating steps.

#### 2.2.3 Enzyme-Assisted Alkali Extraction

In this approach, the method of Latif and Anwar^46^ was followed with a slight modification. First, pumpkin seed flour was mixed with water at a ratio of 1:10 (w/v). The pH of the suspension was set to 8 (optimum working condition of the enzyme) by using 1 M NaOH. Then, the enzyme (Alcalase, 2.4 L) was put into the suspension at a rate of 2% of the sample weight in the enzyme. The preheating steps were applied in this stage. The solutions were shaken in an orbital shaker (Daihan Scientific Co., Ltd., Korea) for 1 h at 100 rpm and then centrifuged at 2263*g* for 15 min. The supernatant was collected, and its pH was adjusted to 11 for alkali treatment. The solution was stirred again for 1 h and centrifuged at 2263*g* for 15 min. The resulting supernatant, containing soluble proteins, was adjusted to pH 5 (the isoelectric point of the protein) by using 1 M HCl to precipitate the proteins. The precipitated proteins were then recovered by centrifugation at 2263*g* for 15 min. Finally, the neutralization and lyophilization steps were carried out as previously described. For the extraction of untreated samples, the same procedure was followed, with the preheating steps omitted.

### 2.3 Analyses

#### 2.3.1 Characterization Analyses

##### 2.3.1.1 Extraction Yield

The equation below was used to calculate the extraction yield (%):
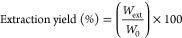
1

Where *W*_ext_ is the weight of the extracted protein (g) obtained after the extraction process

and *W*_0_ is the initial weight (g) of the dried raw pumpkin seed flour used before extraction.

This formula calculates the percentage of the protein extracted from the raw material compared to the total initial weight of the dried sample.

##### 2.3.1.2 Proximate Composition Analysis

Proximate composition analysis of the pumpkin seed flour before and after extractions was conducted for moisture contents and macronutrients (ash, carbohydrates, fat, and protein) by utilizing the AACC Methods (AACC, I., 2000).

An IR moisture balance (also known as an infrared moisture balance) was used for the dried samples for moisture content determination (Radwag MAC 50 Moisture Analyzer, Poland). The data were presented as percentages for all of the analyses.

The method of Zhao and Zhang^47^ was followed to measure the fat contents of the samples. The Soxhlet apparatus (EFLAB) was used to extract the powdered samples using hexane as the solvent.

The modified version of the Kjeldahl method was used to evaluate the total protein amount of the samples by *N* × 6.25.^48^ Finally, total carbohydrate amounts were calculated by following the formula below:
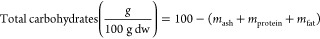
2

##### 2.3.1.3 Fourier Transform Infrared (FTIR) Spectroscopy Analysis

For the analysis, an IR Affinity-1 Spectrometer (Shimadzu Corporation, Kyoto, Japan) with an Attenuated Total Reflectance (ATR) attachment was employed to analyze the powder form of control and extracted samples. Thirty-two scans with a resolution of 16 cm^–1^ were conducted in the 4000–500 cm^–1^ range. The acquired spectra were contrasted with one another and with previously published studies.

The secondary structures of the control (purchased pumpkin seed flour) and extracted PSPC samples were investigated further through quantitative measurement of the Amide I band (1600–1700 cm^–1^). Using the Savitsky–Golay function, OriginPro (2019b, OriginLab Corporation, Northampton, USA) was utilized to process the spectra. By doing a second derivative spectrum analysis, overlapping components were found. The Gaussian function produced the best fit, and 15 points of the window in the positive direction were chosen.^49^

#### 2.3.2 Physicochemical Analyses

##### 2.3.2.1 Soluble Protein Content by Lowry Method

For the analysis, the Lowry method^50^ was followed. 0.5 mL of the sample (1% (w/v) protein solution) with 2.5 mL of Lowry reagent was mixed and then the mixture was left to stand at 25 °C for 10 min. Following this, 0.25 mL of Folin–Ciocalteu’s phenol reagent was put in the tubes, stirred, and left for 30 min in a dark setting. Finally, a UV/vis Spectrophotometer (Optizen POP, South Korea) was used to read the absorbance values at 750 nm. By division of the initial protein content in the samples, the results were reported as percentages.

##### 2.3.2.2 Water Solubility Index (WSI)

WSI was determined by the modified version of the method.^51^ First, the samples were dissolved in distilled water with a 1:4 (w/w) ratio and then put into a shaker (Daihan Scientific Co., Ltd., Korea) at 300 rpm for 1 day to attain complete hydration. Later, the solutions of the samples were centrifugated at 2263*g* for 20 min. The supernatant and sediment were separated, and their weights were measured. The following equation was calculated for the WSI:
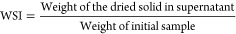
3

##### 2.3.2.3 Hydration Behavior by TD-NMR Relaxometry

The same sample-distilled water ratio (1:4) that was chosen for the WSI experiment was also prepared for the TD-NMR Relaxometry experiment. A 20.34 MHz (0.5 T) NMR instrument (Spin Track, Resonance Systems GmbH, Kirchheim Teck, Germany) was utilized for the analysis to measure T_2_ relaxation times. The Carr–Purcell–Meiboom–Gill (CPMG) pulse sequence was chosen, and 500 ms, 300–500 ms, and 4 were chosen as the echo time, echo number, and number of scans, respectively. To find T_2_ times, MATLAB (R2019b, The MathWorks Inc., USA) was performed to evaluate the monoexponential behavior.

##### 2.3.2.4 Emulsifying Activity (EA) and Emulsifying Stability (ES)

The EA and ES properties were found using the method of Gao et al.^52^ with slight modifications. The 2 mL portion of corn oil and sample solution (8 mL, 0.01 g/mL) were mixed and then homogenized at 20 000 rpm for 2 min. Next, 50 μL of emulsion was taken from the bottom part at 0 and 10 min and diluted with SDS solution (5 mL, 0.1%). The absorbance values of the samples were read at 500 nm by using a UV–vis spectrophotometer (Optizen POP Nano Bio, Mecasys Co. Ltd., South Korea). The absorbance value (A0) was immediately measured after emulsification, and the absorbance value (A10) was measured after 10 min. Finally, the EA and ES values were calculated using the following equations:
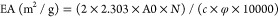
4
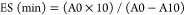
5in which *c* = sample concentration (g/mL), φ = the oil volume ratio of the emulsion (0.25), *N* = dilution factor (101), and A0 and A10 were the absorbance values at 0 and 10 min, respectively.

The experiment also included chicken egg yolk (EY) as a positive control.

##### 2.3.2.5 Foaming Capacity (FC) and Foaming Stability (FS)

The foaming properties (FC and FS) were evaluated by modifying the method of Yang et al.^53^ For the experiment, 1 g of the sample was dissolved in a phosphate solution (0.2 mol/L, pH = 7.4). Next, the 20 mL mixture was homogenized at 20 000 rpm for 2 min. The volume of the foam was measured at 0 and 30 min after homogenization. Finally, the following equations were used to calculate the FC and FS.
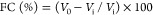
6
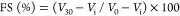
7in which *V*_0_ and *V*_30_ are the foam volumes at 0 and 30 min after homogenization and *V*_i_ is the initial volume before foaming.

Chicken egg white was also used as a positive control in the experiment.

##### 2.3.2.6 DPPH Scavenging Activity

DPPH free radical scavenging activities of samples were determined by following the method of Kim et al.^54^ for the antioxidant activity of the samples. 100 μM DPPH was dissolved in 80% aqueous methanol. 0.1 mL of sample solutions was put into 2.9 mL of the methanolic DPPH solution. Then, the mixture was shaken and left in the dark for 30 min. The decrease in absorbance values was measured at 517 nm for 30 min. For the control, 0.1 mL of 50% aqueous methanol and 2.9 mL of a DPPH solution were prepared and used. The scavenging activity (%) was calculated as
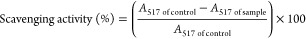
8where *A*_517 of control_ is the absorbance containing only methanol and DPPH solution and *A*_517 of sample_ is the absorbance of sample and DPPH solution.

##### 2.3.2.7 Water Activity (*a*_w_) and Color Analysis

The water activity (*a*_w_) of the extracted proteins was measured by using a water activity meter (AQUALAB 4TE; Aqualab, Pullman, WA, USA).

To determine the color of the samples, a portable spectrocolorimeter (Serlab SL400, Istanbul, Turkey) was used to determine lightness (L*), red-green (a*), and blue-yellow (b*) values.

### 2.4 Statistical Analysis

ANOVA was used to examine the effect of factors on the outcomes of a general linear model regression technique using MINITAB (Version 19, Minitab Inc., Coventry, UK). Tukey’s comparison test with a 95% confidence interval was used to assess significance when needed. The different letters in the figures and tables indicate a significant difference between the samples (*p* < 0.05). Each trial was repeated in triplicate.

## 3 Results and Discussion

### 3.1 Extraction Yield of Pumpkin Seed Protein Concentrate

Many factors influence protein yield extraction, including the type of protein, sample preparation methods, temperature, pH, and the use of enzymes.^55^ In this study, the temperature of preheat treatments was chosen as 50 °C because it was the best option determined by preliminary experiments in the range between 30 and 60 °C. The preliminary results of the temperature range studied for AE samples are shown in Table S1.

Table 1 displays the results for the extraction yield, and according to the results, the highest yield among the extraction techniques was observed in the AE followed by EE and SE samples (*p* < 0.05). Each method (AE, SE, and EE) has its advantages and limitations.^56^ Since proteins are more readily soluble in alkaline environments, alkali extraction frequently yields more protein; however, the high pH can cause the denaturation of proteins and the loss of their functional properties.^57^ A basic solution, such as sodium hydroxide (NaOH), is commonly used to solubilize proteins in alkali extraction to extract both hydrophobic and hydrophilic proteins.^58^ Protein functionality is preserved using enzyme-assisted extraction, which works in milder circumstances but may result in a lower yield, and the cost of enzymes can be a limitation.^59^ Salt extraction efficiently extracts salt-soluble proteins while maintaining their natural structure without harsh chemical treatments. However, to maximize extraction, precise control over ionic strength may be necessary.^60^ It precipitates and isolates proteins using a salt concentration, such as NaCl; nevertheless, salt extraction can precipitate nontarget proteins and may be less successful for hydrophobic proteins.^61^ This might be the reason for having the lowest yield of PSPC among the extraction techniques. Enzyme-assisted alkali (EE) protein extraction uses enzymes, such as proteases, and is commonly used for isolating specific proteins or protein subunits.^62^ When compared with AE samples, EE samples gave lower yields (*p* < 0.05). This could be explained by the fact that the enzyme may not be a target enzyme specifically for PSPC.

**Table 1:** Extraction Yield (% (w/w)) of Pumpkin Seed Protein Concentrate (PSPC) Samples^a^

Treatment	Extraction Techniques	Extraction Yield (% (w/w))
UT	Alkali	34.1 ± 0.05^d^
CH		50.3 ± 0.09^b^
MH		55.2 ± 0.21^a^
UT	Salt	11.2 ± 0.06^h^
CH		14.3 ± 0.07^g^
MH		16.2 ± 0.05^f^
UT	Enzyme-assisted	26.3 ± 0.06^e^
CH		34.5 ± 0.19^d^
MH		36.6 ± 0.26^c^

a UT (untreated samples), CH (conventional heated), and MH (microwave heated). Upper case superscript letters (a–h) denote a significant difference at 5% (*p* < 0.05). Values are expressed as mean ± SE (*n* = 3).

When the preheat treatments were examined, the highest extraction yield was obtained in MH followed by CH and UT, respectively (*p* < 0.05). Temperature is a crucial factor that affects the yield of protein extraction. As the temperature increases moderately, the yield generally increases as well.^63^ This fact compiles well with the results of having the lowest yield in UT samples (*p* < 0.05). Besides, the result of MH having the highest extraction yield can be due to the higher interaction of microwaves with the polar molecules in the extraction media and its working mechanism. In the working principle of the microwave, heat is generated inside the material, and the internal pressure of the solid material is increased spontaneously.^64^ The increased internal pressure may lead to the breakdown of the molecular bonds between the materials, which makes it easier to extract the desired components. In addition, as the material disintegrates, more surface area of the product may be exposed, and this contributes to better contact between the material and surrounding solvent, resulting in higher extraction yields.^65^ A similar trend of higher protein yields in MH extracts compared to CH was reported by Suwannasopon et al.^66^ in their study on soybean protein extraction. Overall, the best combination to get the highest PSPC yield in this design of experiment was found to be in the MH-AE samples (*p* < 0.05).

### 3.2 Proximate Composition Analysis

The results of the samples obtained by different techniques and treatments are reported in Table 2. Besides, the purchased pumpkin seed flour was also analyzed, and it had 9.41 ± 0.03 (g/100 g dw) moisture, 5.44 ± 0.01 (g/100 g dw) ash, 15.5 ± 0.03 (g/100 g dw) fat, 45.13 ± 0.19 (g/100 g dw) protein, and 34.65 ± 0.23 (g/100 g dw) carbohydrate contents.

**Table 2:** Proximate Composition Analysis of the Extracted Pumpkin Seed Protein Concentrate (PSPC) Samples^a^

Treatments	Extraction Techniques	Moisture (g/100 g dw)	Ash (g/100 g dw)	Fat (g/100 g dw)	Protein (g/100 g dw)	Carbohydrate (g/100 g dw)
UT	Alkali	9.24 ± 0.03^a^	5.37 ± 0.01^abcd^	13.41 ± 0.02^c^	59.42 ± 0.11^ef^	21.82 ± 0.33^b^
CH		8.67 ± 0.02^d^	5.60 ± 0.01^a^	11.61 ± 0.03^f^	68.67 ± 0.53^b^	14.14 ± 0.14^e^
MH		7.95 ± 0.02^f^	4.95 ± 0.02^e^	10.63 ± 0.03^g^	76.95 ± 0.52^a^	7.51 ± 0.06^f^
UT	Salt	8.23 ± 0.03^e^	5.23 ± 0.02^cd^	15.19 ± 0.07^ab^	55.23 ± 0.84^g^	24.44 ± 0.45^a^
CH		8.72 ± 0.03^cd^	5.12 ± 0.05^de^	15.22 ± 0.06^a^	61.22 ± 0.59^de^	18.44 ± 0.23^c^
MH		8.55 ± 0.06^d^	5.55 ± 0.05^ab^	14.91 ± 0.06^b^	63.55 ± 0.74^cd^	15.98 ± 0.41^de^
UT	Enzyme-assisted	9.13 ± 0.04^ab^	5.13 ± 0.07^de^	13.10 ± 0.06^d^	57.13 ± 0.65^fg^	24.64 ± 0.44^a^
CH		9.02 ± 0.03^b^	5.42 ± 0.09^abc^	12.72 ± 0.03^e^	63.52 ± 0.50^cd^	18.36 ± 0.26^c^
MH		8.93 ± 0.04^bc^	5.3 ± 0.03^bcd^	10.80 ± 0.05^g^	66.93 ± 0.63^bc^	16.97 ± 0.15^cd^

a UT (untreated samples), CH (conventional heated), and MH (microwave heated). Values are expressed in dry weight (dw) as mean ± SE (*n* = 3). Upper case superscript letters (a–g) denote a significant difference at 5% (*p* < 0.05) in each column.

When comparing these results with all results in Table 2, it was shown that the protein content is the lowest and the carbohydrate content is the highest in the purchased flour (*p* < 0.05). This demonstrated that, regardless of the method or treatment used in the extraction procedure, all samples were successfully extracted.

In Table 2, the moisture content values were found to range from 8.23 to 9.24 (g/100 g dw), indicating that different methods and treatments had affected the moisture content (*p* < 0.05). The determination of moisture content is important in food components because it affects how long the food will last and how storage conditions should be decided.^67^ Furthermore, it can play a crucial role in the hydration behavior of food components.^68^

The results for the ash contents ranged from 4.95 to 5.60 (g/100 g dw). According to ANOVA results that compared extraction techniques, the results did not show significant differences (*p* > 0.05). However, different treatments were shown to be significantly different in the samples (*p* < 0.05). When all results were compared, there were some differences, in which the highest ash content belonged to the CH-AE sample (*p* < 0.05). The higher ash content in the CH-AE sample could be attributed to the effect of conventional heating during alkali extraction, which may enhance the release of minerals and inorganic compounds from the plant matrix due to prolonged heat exposure.^69^

Table 2 shows that the fat contents ranged from 10.64 to 15.22 (g/100 dw). The comparison of the extraction techniques showed that the SE samples had the highest amount of fat content followed by EE and AE samples (*p* < 0.05). Besides, between the treatments, UT samples had the highest fat contents followed by MH and CH treatments, respectively (*p* < 0.05). The fact that the amount of fats in PSPC samples did not decrease significantly following extractions can be evaluated as an advantage since fats are a source of essential or nonessential fatty acids, antioxidants, and energy.^70^

When the total protein contents were examined in Table 2, the range was found to be from 55.23 to 76.95 (g/100 dw). The purchased native pumpkin seed flour had the lowest protein content at 45.13 ± 0.19 (g/100 g dw). Therefore, they were not put into the statistical analysis and Table 2 since it would not be easy to see the differences between the data sets of the obtained samples. This outcome was expected since the aim of this study was to achieve the extraction of proteins. Between the extraction techniques, it was seen that AE samples gave the highest protein content followed by EE and SE samples, respectively (*p* < 0.05). In the literature, it was shown that the increase in pH up to a certain value (∼11–12) increased the protein amount diffused into solutions, hence the higher contents of the protein after the extraction,^71^ which was also confirmed in our study. When the effect of preheat treatment was compared, MH samples showed the highest protein contents followed by CH and UT samples, respectively (*p* < 0.05). The lowest yield of UT can be linked to the effect of temperature since a moderate increase in temperature may contribute to an enhancement of the protein yield.^63^

In our results, the best combination to get the highest protein content was found to be in the MH-AE samples, with a value of 76.95 ± 0.52 (*p* < 0.05). Microwave energy increases the rate of diffusion, allowing for the extraction of proteins from the sample at a faster rate.^64^ Moreover, microwave use can also cause mechanical forces such as pressure to be generated, which can help matrix disruption and protein release.^72^ The overall effect therefore might have been higher protein yield compared with traditional methods with the combination of heat, pressure, and solvent extraction.

The carbohydrate contents were found to range from 7.51 to 24.64 (g/100 dw). The original pumpkin seed flour had the highest amount at 34.65 ± 0.23 (g/100 g dw), but they were not evaluated for the statistical analysis and Table 2 again due to being the outliers for the data set.

The results obtained from carbohydrates are negatively correlated with the protein content results. For instance, the lowest carbohydrate content was seen in the MH-AE samples (*p* < 0.05). Some insoluble components in the carbohydrates such as dietary fibers and cellulose can cause molecular crowding in the solution,^73^ and this can cause lower protein–water interaction and lower solubility of proteins. Therefore, the proteins in the MH-AE samples, having the lowest carbohydrate content, are likely to be more freely available in solution and, as a result, potentially more functional.

### 3.3 Fourier Transform Infrared (FTIR) Spectroscopy

**Figure 1:**
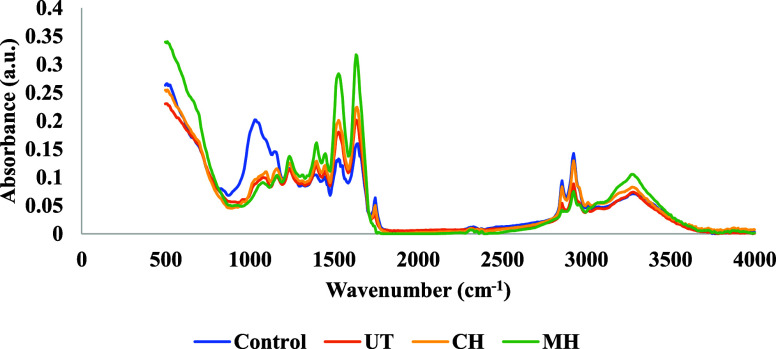
Fourier-transform infrared spectroscopy (FTIR) spectra of the control (purchased pumpkin seed flour) and alkali extracted (AE) pumpkin seed protein concentrate (PSPC) samples.

It is a widely used technique for the identification of functional groups and structural changes in the compounds,^74^ and this study demonstrated structural differences in extracted samples. In Figure 1, the FTIR spectra of AE samples are given. The spectra obtained for EE and SE were supplied separately in Figures S1 and S2. Besides, the purchased pumpkin seed flour was also examined and given as the control in Figure 1.

When the spectra were investigated, the peak corresponding in the range of 1000–1100 cm^–1^ was correlated with the coupling of the C–O or the C–C stretching bands.^75^ The component’s relative carbohydrate content may be estimated from the intensity of this peak; thus, the highest intensity can be related to having a high amount of carbohydrates. In the figure, the peak of the control sample (pumpkin seed flour) was much higher compared to that of the extracted ones. Besides, the decrease in MH samples was much more than in other samples. This observation can also be supported by the carbohydrate contents obtained in the proximate composition analysis section.

The observed peaks of 2856 and 2927 cm^–1^ with C–H stretching of −CH_3_, −CH_2_ provide information about the fat contents of compounds,^76^ and as in the figure, while the control sample had the highest peak, the lowest belonged to MH samples. Again, fat contents in the proximal analysis are consistent with these findings.

When it comes to observing the peaks related to proteins, it was seen that literature is generally focused on two of the most crucial peaks, Amide I (∼1700–1600 cm^–1^) and Amide II (∼1585–1480 cm^–1^) bands.^77^ In these bands, changes in the C=O stretching of the Amide I band can be used to assess the secondary structures of the proteins, and the C–N stretching vibrations and N–H bending of the Amide II band are utilized to monitor the conformational sensitivity and unfolding of the proteins.^78^ In the protein extractions, an increase was expected in those peaks because of more protein content in the matrix after the extraction procedure. According to the figure, the highest peaks were observed in MH-AE samples, but the lowest peaks belonged to the control samples. These outcomes showed that the indication of extraction and FTIR results are in accordance with the other results.

The secondary structures of control (purchased pumpkin seed flour) and extracted PSPC samples were further identified by analyzing the derivative spectra in the Amide I region (1700–1600 cm^–1^), and the results for AE samples are shown in Table 4. The results for SE and EE samples are given in Tables S2 and S3.

In the analysis, four peaks were observed as α-Helix (1648–1657 cm^–1^), β-sheet (1612–1641 cm^–1^), β-turn (1660–1684 cm^–1^), and random coil (1640–1650 cm^–1^) as shown also in other studies.^79^ When these contents were examined, it was seen that α-Helix and β-sheet were the predominant structures, which is consistent with the findings of previous research.^80,81^ Besides, within the different extraction approaches, it was observed that α-Helix contents decreased, whereas β-sheet content increased independent of the treatments applied (*p* < 0.05). Indeed, the lowest content belonged to the MH-treated samples followed by CH and UT (*p* < 0.05). Similar results were also obtained for the SE and EE samples. Although the effect of extraction techniques on secondary structures of pumpkin seed protein is not well studied, a study on bovine serum albumin (BSA) found a similar pattern.^80^ The study showed that the β-sheet content increased, while the α-helix content decreased because of the changes in different extraction processes. Also, the changes because of the extraction approaches might have a significant impact on protein–water interactions, primarily due to alterations in hydrogen bonding. It was stated in the studies that the α-Helix structure is often more compact and might form stable structures,^82^ which can lower protein–water interaction. In contrast, the β-sheet structure might have more exposed hydrophilic surfaces due to their extended nature,^83^ which can allow more protein–water interaction.

### 3.4 Protein Solubility, WSI, and Hydration Behavior

Pumpkin seed flour, like many other plant-based flours, has a lower protein solubility issue due to being large and complex molecules that can make it difficult to diffuse proteins into the solution. Besides, protein solubility is affected by several factors, including the structure of the protein, temperature, interaction with other molecules, salts, and extraction methods from their native forms.^77^ Since extraction methodology plays a crucial role in protein–water interaction and, thereby, solubility, this study focused on the examination of this phenomenon. For that, related studies such as protein solubility, WSI, and T_2_ relaxation times were performed and are shown in Table 3.

**Table 3:** Fourier-Transform Infrared Spectroscopy (FTIR) Spectra of Control (Purchased Pumpkin Seed) and Alkali-Extracted (AE) Pumpkin Seed Protein Concentrate (PSPC) Samples^a^

Samples	α-Helix (%)	β-Sheet (%)	β-Turns (%)	Random Coil (%)
Control	36.26 ± 0.42^a^	37.46 ± 0.63^d^	17.27 ± 0.12^c^	8.99 ± 0.02^c^
UT	32.41 ± 0.38^b^	40.36 ± 1.13^b^	17.35 ± 0.12^c^	9.87 ± 0.02^b^
CH	31.99 ± 0.34^c^	39.76 ± 0.66^c^	18.22 ± 0.44^b^	10.02 ± 0.12^a^
MH	29.13 ± 0.22^d^	40.81 ± 0.54^a^	20.04 ± 0.47^a^	10.01 ± 0.11^ab^

a Control (Purchased Pumpkin Seed Flour), UT (untreated samples), CH (conventional heated), and MH (microwave heated). Values are expressed as mean ± SE (*n* = 3). Upper case superscript letters (a–d) denote a significant difference at 5% (*p* < 0.05) in each column.

Within the extraction techniques, the highest solubility was found for the samples in the AE followed by EE and SE ones (*p* < 0.05) Table 4. The reason for the lowest solubility of the samples in the SE can be explained by the behavior of the used salt as being “salting out” in the solution. It was stated that if the salts are acting as salting out in the solutions, it can affect the stability of the protein–water interactions, leading to the precipitation of proteins and lower solubility.^84^

**Table 4:** Protein Solubility (PS) (% (w/w)), Water Solubility Index (WSI), and T_2_ Relaxation Times (Milliseconds) of Extracted Pumpkin Seed Protein Concentrate (PSPC) Samples^a^

Treatments	Extraction Techniques	PS (% (w/w))	WSI (w/w))	T_2_(ms)
UT	Alkali	10.32 ± 0.05^c^	3.31 ± 0.009^b^	127.94 ± 0.774^f^
CH		9.75 ± 0.02^d^	2.32 ± 0.03^e^	229.81 ± 1.21^d^
MH		15.99 ± 0.04^a^	3.45 ± 0.005^a^	113.02 ± 1.01^g^
UT	Salt	7.86 ± 0.01^f^	2.59 ± 0.02^d^	355.01 ± 2.24^a^
CH		7.97 ± 0.01^f^	1.99 ± 0.03 ^g^	319.54 ± 2.87^b^
MH		8.04 ± 0.02^f^	2.75 ± 0.007^c^	274.89 ± 1.91^c^
UT	Enzyme-assisted	9.88 ± 0.02^d^	3.29 ± 0.009^b^	164.09 ± 1.19^e^
CH		9.11 ± 0.03^e^	2.11 ± 0.009^f^	228.61 ± 4.03^d^
MH		13.68 ± 0.03^b^	3.41 ± 0.005^a^	125.22 ± 2.78^fg^

a UT (untreated samples), CH (conventional heated), and MH (microwave heated). Values are expressed as mean ± SE (*n* = 3). Upper case superscript letters (a–g) denote a significant difference at 5% (*p* < 0.05) in each column.

Similar results were reported by Wang et al.^85^ in their study on the influence of ionic strength on soy protein solubility, where they showed that higher salt concentrations led to reduced solubility due to protein aggregation with the “salting out” effect. When the results of different heat treatments were examined, the MH samples were found to be solubilized more than the CH and UT samples, respectively (*p* < 0.05). The reason for the lowest solubility of UT samples can be correlated with the temperature effect. Besides, the reason for MH samples having higher solubility than CH may be that microwave heating can extract proteins under milder conditions, which may help to preserve the native conformation of the proteins and prevent denaturation and aggregation.^86^ Additionally, microwave energy can damage the cell membrane, and then release intracellular contents, and solubilize the proteins because of the increased internal pressure effect inside the material.^87^ Within the spontaneous increase in internal pressure, the disintegration of the material would be facilitated, leading to higher extraction yields and more interaction with the water, which would also effectively solubilize more of the proteins. A study by Varghese et al.^88^ demonstrated similar results in soymilk protein that microwave heating improved protein solubility compared to conventional heating methods with more extraction yield. Besides, as given in the proximate composition analysis part, MH samples had the lowest carbohydrate contents but the highest protein contents. Having fewer carbohydrates in the solution may lead to more protein–water interactions in the solution, thereby increasing the soluble protein content. Overall, the extraction method employed significantly influenced the protein solubility and, by extension, other functional properties.

Analyzing the WSI results is another method to see the water interaction. WSI increases as the soluble contents diffuse into the water.^51^ Considering the results obtained from proximate composition analyses, we can say that the main contributor to the soluble portions is expected to be proteins in our extracted samples. According to the results, the highest WSI values were found for AE followed by SE and EE between the techniques, respectively (*p* < 0.05). In addition, for the heat treatments, MH samples had the highest results followed by CH and UT samples (*p* < 0.05). These outcomes also match with the solubility results obtained by the Lowry method. Thus, it can be claimed that both the solubility and WSI results are interrelated, and this claim is corroborated by the Pearson correlation between solubility and WSI with a correlation coefficient of 0.704 (*p* < 0.05).

The water interaction of the solutes may further be determined by T_2_ relaxation times. These times may supply information regarding the dynamics of water (mobile/immobile or bound/free) in a food system.^89,90^ If this needs to be interpreted, longer T_2_ times are associated with more free water in the system. In our case, we can evaluate the results as if we had more soluble protein in the solution, we would have shorter T_2_ times due to protein–water interactions. According to Table 4, the T_2_ relaxation times of the extracts were found significantly different (*p* < 0.05). Moreover, there is a strong negative correlation between the WSI and T_2_ relaxation times with a correlation coefficient of −0.780 (*p* < 0.05). This is expected since WSI is related to more soluble contents, which would result in less free water in the solution and, thus, shorter T_2_ relaxation times. For instance, in the results, the highest WSI value was seen for MH-AE samples (*p* < 0.05). When T_2_ relaxation times were looked for in MH-AE samples, it was observed that the T_2_ times were the shortest (*p* < 0.05). The same negative correlation was also seen for the other results between the WSI and T_2_ relaxation times. Therefore, it was deduced that TD-NMR relaxometry can be effectively utilized to investigate protein–water interactions.

### 3.5 Emulsifying Activity (EA) and Emulsifying Stability (ES)

**Figure 2:**
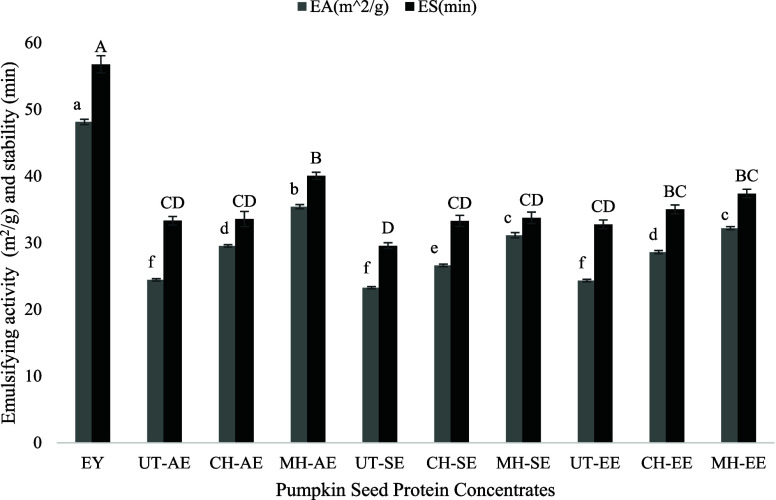
Emulsifying activity (EA) (m^2^/g) and emulsifying stability (ES) (min) of egg yolk (EY) and extracted pumpkin seed protein concentrate (PSPC) samples.

The EA and emulsion stability ES of PSPC samples were compared with EY as a control, and the results are given in Figure 2.

EY, a well-known emulsifier, contains a phospholipid called lecithin, which improves the interaction between the water and oil phases in emulsions and is primarily responsible for better emulsifying characteristics.^91^ Because lecithin is amphiphilic, it can efficiently stabilize emulsions by lowering surface tension and forming a protective layer around oil droplets.^92^

According to the results, EY showed the highest EA and ES significantly among all samples (*p* < 0.05). When the results were compared between PSPC samples, it can be concluded that EA increased with the application of preheat treatments (*p* < 0.05). The highest EA was observed for MH-treated samples (*p* < 0.05). Between the extraction techniques, the EA of the samples was the highest in AE followed by EE and SE, respectively (*p* < 0.05). Several factors could support the reason. More soluble proteins in the solution may lead to the diffusion of more hydrophilic groups, which could enhance the interaction between the proteins and the oil, producing better EA and ES.^93^ Besides, in previous studies, it was observed that secondary structures of the proteins play a role in the EA and ES of a protein.^94,95^ Specifically, higher random coil content in a protein has been linked to improved EA and ES.^94^ This is because random coils provide more flexibility^96^ that could allow proteins to more easily interact with oil droplets. These outcomes also align with the results obtained through solubility and FTIR results. Although the PSPC samples exhibited lower EA and ES values than EY, the results are still promising, especially for MH-AE. The findings showed potential for developing PSPC as a functional emulsifier in the food industry, providing a beneficial plant-based substitute for EY.

### 3.6 Foaming Capacity (FC) and Foaming Stability (FS)

**Figure 3:**
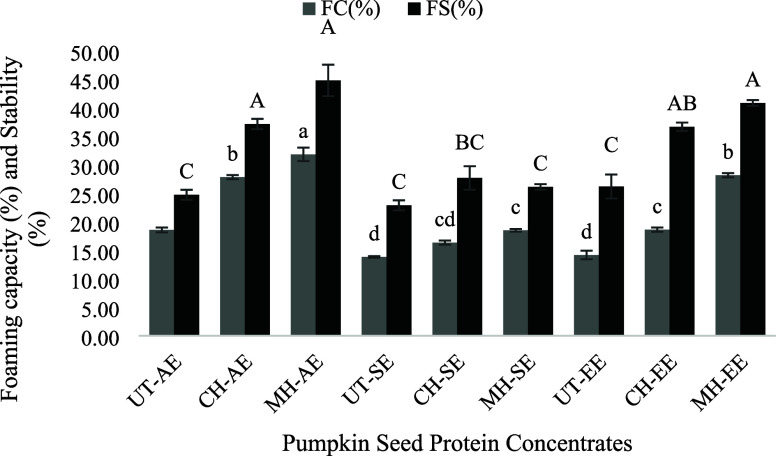
Foaming capacity (FC) (%) and foaming stability (FS) (%) of extracted pumpkin seed protein concentrate (PSPC) samples.

The egg white was included as a positive control in this study since it has well-established foaming properties, which are frequently cited in the literature.^97,98^ The measured values for egg white, with FC of 135.7 ± 1.75% and FS of 70.06 ± 0.68%, align with those in the literature.^99,100^ However, the results were not incorporated into the statistical analysis since the significant difference between the egg white and extracted PSPC samples would have caused data skewing, making the comparison insignificant. The egg white was therefore only employed as a reference point, and its remarkable outcomes confirmed what was expected based on the literature. For the extracted PSPC, the FC and FS are represented in Figure 3.

The results showed that pretreated samples increased both FC and FS, with the highest properties seen in MH, CH, and UT(*p* < 0.05). Additionally, as seen with emulsifying properties, AE samples gave better results among the extraction techniques (*p* < 0.05). The highest FC and FS (by mass) were obtained from the samples obtained by the combined effect of MH and AE (*p* < 0.05). This can be attributed to both the higher protein content and the improved solubility of the extracted proteins. Increased protein concentration enhances the ability of proteins to form and stabilize foams,^101^ while with increased solubility, hydrophilic groups and their diffusion rate to air–water interfaces may enhanced, which would result in stronger foaming properties.^102^ In addition, the effect of heat treatment to enhance foaming properties was explained in previous studies that a drop in viscosity of the solution because of heating increases the penetration in the compounds, which could facilitate obtaining more improved foaming properties.^103^ Furthermore, improved alignment of proteins at the air–water interface and increased protein flexibility as a result of heat treatment and extraction methods can improve the capacity of proteins to form stable foams.^104^

### 3.7 Antioxidant Activity

The antioxidant activity (AA) of the extracted PSPC samples was determined and is shown in Table 5.

**Table 5:** Antioxidant Activity (AA) (mg Trolox/g Sample) of Extracted Pumpkin Seed Protein Concentrate (PSPC) Samples^a^

Treatments	Extraction Techniques	AA (mg Trolox/g sample)
UT	Alkali	15.24 ± 0.02^b^
CH		14.63 ± 0.07^e^
MH		15.54 ± 0.08^a^
UT	Salt	14.56 ± 0.02^f^
CH		14.12 ± 0.09^ı^
MH		14.39 ± 0.06^h^
UT	Enzyme-assisted	15.08 ± 0.03^c^
CH		14.44 ± 0.04^g^
MH		14.72 ± 0.03^d^

a UT (untreated samples), CH (conventional heated), and MH (microwave heated). Values are expressed as mean ± SE (*n* = 3). Upper case superscript letters (a–g) denote a significant difference at 5% (*p* < 0.05) in each column.

It is known that heating could cause a decrease in antioxidant activities,^105^ and with the preheat treatment in our experiments, there was a chance to degrade the antioxidant activities of the extracted PSPC samples. According to the results, it can be said that the samples that were exposed to CH had decreased the AA of samples (*p* < 0.05), which can be linked to the longer duration of heating to obtain the extracted PSPC.^73^ Besides, the highest AA was obtained for MH-AE samples (*p* < 0.05). This can be seen as the advantage of microwave heating. Due to shorter exposure of time, the degradation of AA would be eliminated or even higher AA could be obtained, thanks to more soluble proteins in the solution.^106^ To make the proteins more functional, it is important to preserve their AA, and it has been shown in this study that MH pretreatment can be a suitable choice. Proteins with high AA can help prevent lipid oxidation and preserve the quality and shelf life of food products by neutralizing free radicals.^107^ For this reason, they can be beneficial in meat, dairy, and emulsion products where oxidation can degrade the nutritional value, flavor, and texture.^108^ Studies have shown that the MH pretreatment effectively preserved the AA of the proteins^106,109^ as also shown in our study.

### 3.8 Water Activity (*a*_w_) and Color Analysis

The water activity (*a*_w_) and color analysis of the PSPC samples were measured and are given in Table 6.

**Table 6:** Water Activity (*a*_w_) and Color Analysis (L*, a*, and b*) of Extracted Pumpkin Seed Protein Concentrate (PSPC) Samples^a^

Treatments	Extraction Techniques	Water Activity (*a*_w_)	L*	a*	b*
UT	Alkali	0.31 ± 0.005^a^	49.57 ± 0.07^c^	4.13 ± 0.03^b^	28.77 ± 0.03^c^
CH		0.32 ± 0.005^a^	45.17 ± 0.03^g^	4.27 ± 0.03^ab^	27.47 ± 0.06^d^
MH		0.31 ± 0.003^a^	48.67 ± 0.07^d^	4.13 ± 0.02^b^	28.57 ± 0.02^c^
UT	Salt	0.30 ± 0.005^a^	51.27 ± 0.07^a^	3.33 ± 0.03^d^	30.23 ± 0.03^a^
CH		0.32 ± 0.002^a^	48.77 ± 0.03 ^d^	3.73 ± 0.02^c^	29.80 ± 0.05 ^b^
MH		0.31 ± 0.003^a^	50.37 ± 0.03^b^	3.43 ± 0.03^d^	29.97 ± 0.05^ab^
UT	Enzyme-assisted	0.33 ± 0.003^a^	47.57 ± 0.08^e^	4.27 ± 0.02^ab^	26.73 ± 0.05^e^
CH		0.32 ± 0.005^a^	46.77 ± 0.06^f^	4.43 ± 0.03^a^	26.53 ± 0.03^e^
MH		0.32 ± 0.006^a^	47.46 ± 0.02^e^	4.20 ± 0.05^b^	26.70 ± 0.05^e^

a UT (untreated samples), CH (conventional heated), and MH (microwave heated). Values are expressed as mean ± SE (*n* = 3). Upper case superscript letters (a–g) denote a significant difference at 5% (*p* < 0.05) in each column.

Table 6 showed that the water activity values of the extracted proteins were statistically insignificant and found consistently low, around 0.3, which could indicate good stability.

The last stage of the freeze-drying process to obtain extracts in dry form can help to the maintenance of stable *a*_w_ levels by removing free water, which would suggest good stability against microbial growth and chemical reactions.^110,111^

The color analysis, however, showed slight variations in L*, a*, and b* values across the samples, as shown in Table 6. Lightness is represented by the L* value, where higher values denote a lighter hue and lower values a darker product.^112^ When Table 6 was examined, it was seen that CH led to darker products with lower L* values followed by MH and UT, respectively, between the treatments (*p* < 0.05). This suggested that longer heat exposure in CH treatments may contribute to browning reactions and thereby pigment degradation.^113^ MH, on the other hand, tends to retain the lightness due to shorter exposure times and more localized heating, which can also be confirmed by literature studies that MH is less destructive in food decolorization.^114^ Among the extraction methods, SE preserved the most natural color, with higher L* values, followed by EE and AE, respectively (*p* < 0.05). SE is generally considered a milder process compared to AE and EE methods since it does not involve extreme pH changes or enzyme activity that may disrupt pigment. Hewage et al.^115^ et al. highlighted that at both high and low pH levels, phenolic compounds oxidize into reactive o-quinones or o-dihydroxy structures, which can bind to proteins, leading to the darker coloration of plant protein extracts.

The a* value represents the position on the green-red axis, with positive values denoting redness and negative values denoting greenness.^116^ According to the results, CH resulted in higher a* values (more redness) followed by MH and UT, respectively (*p* < 0.05), which may be again linked to browning reactions with longer exposure to heat treatment. Again, between extraction methods, SE maintained the lowest a* values followed by EE and AE (*p* < 0.05), respectively, indicating less change in natural pigments.

The yellow-blue axis is shown by the b* values, where positive values denote yellowness and negative values denote blueness. Positive b* values indicate that all of the study’s samples are more yellow than blue. Between the treatments, CH caused the most decrease in b* values (less yellowness), followed by MH and UT, respectively (*p* < 0.05). In the extraction methods, EE showed the lowest b* values, possibly due to enzyme-induced exposure of pigments to degradation or oxidation followed by AE and SE, respectively (*p* < 0.05).

Overall, SE best retained the lightness (L*) and natural yellow color (b*) while contributing the smallest increase in redness (a*) compared to AE and EE methods. Furthermore, CH treatment significantly reduced L* and b* values and increased a* values, while MH had a milder effect, better retaining the natural color. Since color is an important factor in food formulation and consumer acceptance, the selection of the optimum extraction approach should be conducted based on the desired properties.^117^

In conclusion, this study highlights the potential of microwave-heated alkali extraction (MH-AE) as an innovative and valuable method for extracting pumpkin seed protein concentrate (PSPC) with an enhanced yield and functional properties. The combination of increased protein yield and improved functionality demonstrates the advantages of this method over conventional methods. In addition to these results, the findings suggest that MH-AE can be further explored for its application in plant-based protein products, especially in industries searching for sustainable and high-quality protein sources. Future research could investigate the scalability of this method and its potential for optimizing protein extractions from other plant sources, extending its application in industry.

## Data Availability

Data are available on request from the authors.

## Supporting Information Available

Tables on the protein yield via alkali extraction through different time–temperature combinations (Table S1), secondary structures of SE and EE methods (Table S2 and Table S3) and their FTIR spectra figures (Figure S1 and Figure S2) (PDF)

## Abbreviations

AE: Alkali extracted
ANOVA: Analysis of variance
CH: Conventional heated
CH-AE: Conventional heated alkali extracted
CH-EE: Conventional heated enzyme-assisted extracted
CH-SE: Conventional heated salt extracted
EE: Enzyme-assisted alkali extracted
FTIR: Fourier transform spectroscopy
MAE: Microwave-assisted extraction
MH: Microwave-heated
MH-AE: Microwave-heated alkali extracted
MH-EE: Microwave-heated enzyme-assisted extracted
MH-SE: Microwave-heated salt extracted
PSPC: Pumpkin seed protein concentrate
SE: Salt extracted
TD-NMR: Time-Domain Nuclear Magnetic Resonance
UT: Untreated
UT-AE: Untreated alkali extracted
UT-EE: Untreated enzyme-assisted extracted
UT-SE: Untreated salt extracted
WSI: Water solubility index
