# Supplementary material for: Effect of Extraction Methods and Preheat Treatments on the Functional Properties of Pumpkin Seed Protein Concentrate
Source: ACS Food Sci Technol. 2024 Dec 25;5(1):105–17. doi: 10.1021/acsfoodscitech.4c00601 (PMC11748320; doi:10.1021/acsfoodscitech.4c00601)
Supplement: Supplementary file 1 — fs4c00601_si_001.pdf [file fs4c00601_si_001.pdf]

Supporting Information

Effect of Extraction Methods and Pre-Heat Treatments on the Functional Properties of Pumpkin Protein Concentrate

Ozan Tas[a], S. Gulum Sumnu[a], Mecit Halil Oztop[a*]

[a] Department of Food Engineering, Middle East Technical University, Ankara, 06800, Turkey

***Corresponding author:** mecit@metu.edu.tr

Table of Contents

p.2 Table of Contents

p.3 Table S1 Extraction yield (% (w/w)) of Pumpkin Seed Protein (PSP) samples obtained from the alkali method with different temperature-time combinations.

p.4 Table S2 Fourier-transform infrared spectroscopy (FTIR) spectra of control (purchased pumpkin seed) and salt extracted (SE) Pumpkin Seed Protein Concentrate (PSPC) samples.

p.5 Table S3 Fourier-transform infrared spectroscopy (FTIR) spectra of control (purchased pumpkin seed) and enzyme assisted-alkali extracted (EE) Pumpkin Seed Protein Concentrate (PSPC) samples.

p.6 Fig. S1. Fourier-transform infrared spectroscopy (FTIR) spectra of enzyme-assisted alkali extracted (EE) Pumpkin Seed Protein (PSP) samples.

p.7 Fig. S2. Fourier-transform infrared spectroscopy (FTIR) spectra of salt extracted (SE) Pumpkin Seed Protein (PSP) samples.

Table S1 Extraction yield (% (w/w)) of Pumpkin Seed Protein (PSP) samples obtained from the alkali method with different temperature-time combinations.

Pre-heating Treatments	Temperatures (°C)	Time (min)	Yields (%, w/w)
Water Bath	30 °C	12	31.3±0.14^{d}
	40 °C	13	35.6±0.22^{c}
	50 °C	15	54.4±0.13^{a}
	60 °C	17.5	39.7±0.16^{b}
Microwave	30 °C	0.5	38.3±0.14^{d}
	40 °C	0.7	47.7±0.15^{c}
	50 °C	0.85	66.7±0.23^{a}
	60 °C	1	51.4±0.31^{b}

Upper case superscript letters (a-d) denote a significant difference at 5% ($p<0.05$) in each treatment separately.

Table S2 Fourier-transform infrared spectroscopy (FTIR) spectra of control (purchased pumpkin seed) and salt extracted (SE) Pumpkin Seed Protein Concentrate (PSPC) samples.

**Samples**	**α-Helix (%)**	**β-Sheet (%)**	**β-Turns (%)**	**Random Coil (%)**
Control	36.26± 0.42[a]	37.46±0.63[d]	17.27±0.12[c]	8.99±0.02[c]
UT	35.59±0.27[b]	40.59±0.44[a]	17.45±0.22[b]	9.34±0.32[b]
CH	34.45±0.37[c]	38.45±0.56[c]	18.17±0.35[a]	8.91±0.25[d]
MH	31.11±0.24[d]	39.84±0.43[b]	18.16±0.22[a]	10.88±0.11[a]

Control (Purchased Pumpkin Seed Flour), UT (Untreated samples), CH (Conventional heated), and MH (Microwave heated). Upper case superscript letters (a-d) denote a significant difference at 5% ($P < 0.05$) in the same column. Values are expressed as mean ± SE (n=3).

Table S3 Fourier-transform infrared spectroscopy (FTIR) spectra of control (purchased pumpkin seed) and enzyme assisted-alkali extracted (EE) Pumpkin Seed Protein Concentrate (PSPC) samples.

Samples	α-Helix (%)	β-Sheet (%)	β-Turns (%)	Random Coil (%)
Control	36.26± 0.42^{a}	37.46±0.63^{d}	17.27±0.12^{b}	8.99±0.02^{d}
UT	32.13±0.31^{b}	40.01±0.38^{c}	17.21±0.63^{b}	10.65±0.13^{b}
CH	31.83±0.33^{c}	41.11±0.79^{b}	17.95±0.23^{a}	9.12±0.24^{c}
MH	30.74±0.28^{d}	41.79±0.93^{a}	16.32±0.27^{c}	11.14±0.28^{a}

Control (Purchased Pumpkin Seed Flour), UT (Untreated samples), CH (Conventional heated), and MH (Microwave heated). Upper case superscript letters (a-d) denote a significant difference at 5% ($P < 0.05$) in the same column. Values are expressed as mean ± SE (n=3).

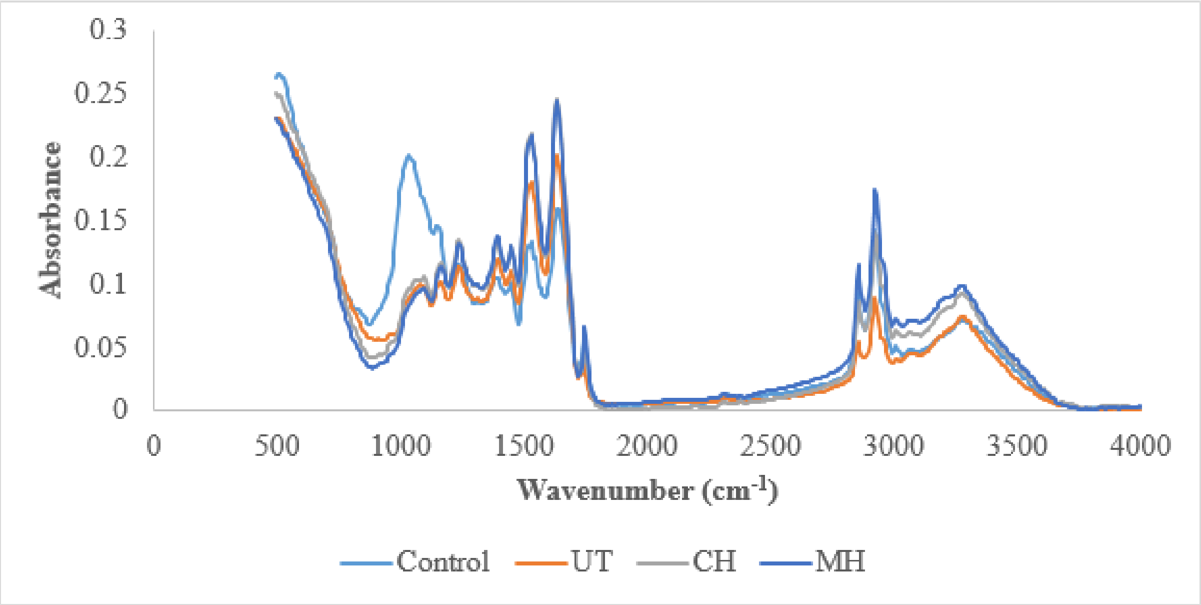

Fig. S1. Fourier-transform infrared spectroscopy (FTIR) spectra of enzyme-assisted alkali extracted (EE) Pumpkin Seed Protein (PSP) samples.

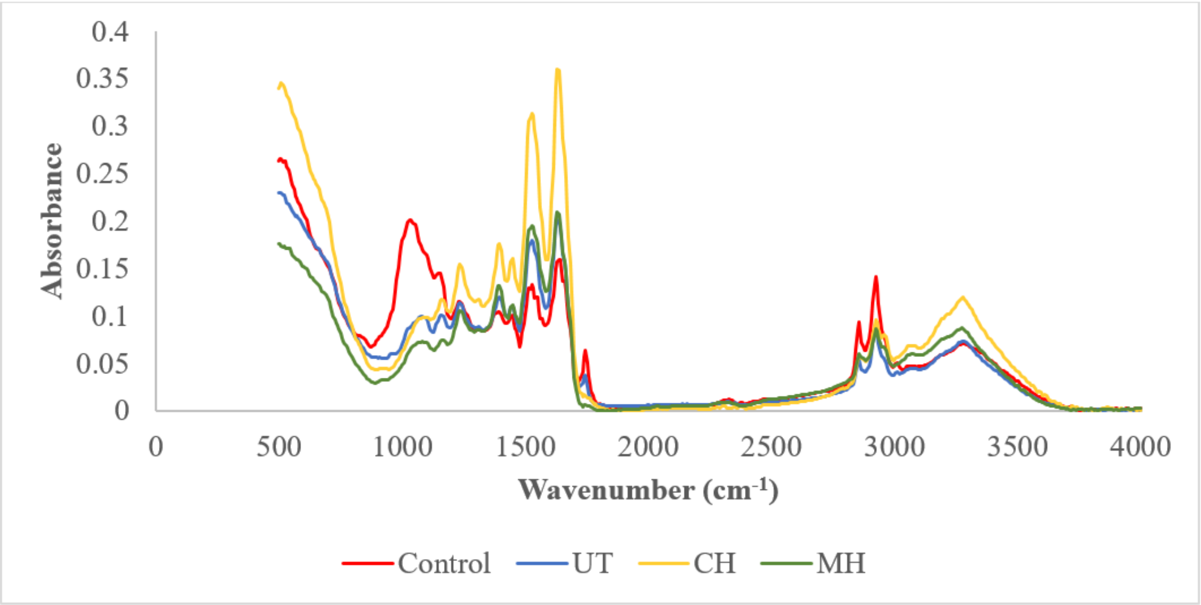

Fig. S2. Fourier-transform infrared spectroscopy (FTIR) spectra of salt extracted (SE) Pumpkin Seed Protein (PSP) samples.
